# Sex-specific associations between pregnancy zone protein and acute coronary syndrome: a case-control study

**DOI:** 10.1186/s13293-026-00926-5

**Published:** 2026-05-22

**Authors:** Xinxin Shen, Xiaobin Guo, Ting Yang, Kaifang Yang, Weilin Wang, Huimin Chen, Fengling Lai, Mingjun Zhang, Yan Zheng, Guohai Su, Keqing Hu, Rong Huang

**Affiliations:** 1 Department of Cardiology, Central Hospital Affiliated to Shandong First Medical University, Jinan, 250013 Shandong China; 2 Medical Science and Technology Innovation Center, Shandong First Medical University and Shandong Academy of Medical Sciences, Jinan, 250013 Shandong China; 3 School of Public Health, Shandong First Medical University and Shandong Academy of Medical Sciences, Jinan, 250013 Shandong China; 4 Research Center of Translational Medicine, Central Hospital Affiliated to Shandong First Medical University, Jinan, 250013 Shandong China

**Keywords:** Acute coronary syndrome, Pregnancy zone protein, Sex differences

## Abstract

**Background:**

Pregnancy zone protein (PZP) is a highly glycosylated macromolecular protein involved in energy metabolism, fibrinolysis, and immunomodulation. However, its association with acute coronary syndrome (ACS), particularly regarding sex differences, remains to be fully elucidated.

**Methods:**

ACS patients were enrolled from the Department of Cardiology, and control subjects were recruited from the Health Examination Department. Plasma levels of PZP were quantified using a sandwich enzyme-linked immunosorbent assay (ELISA). Spearman’s correlation was used to analyze the association between PZP levels and clinical variables. The association of PZP with ACS was assessed using logistic regression models, with PZP levels analyzed as a continuous variable, and subsequently categorized into binary and quartile groupings. Potential confounding variables were selected based on the disjunctive cause criterion and were adjusted for in the logistic regression models.

**Results:**

In this study, we enrolled 1170 participants, comprising 721 men and 449 women. Plasma PZP exhibited marked sexual dimorphism, with concentrations much higher in women than in men (median [IQR]: 3.46[1.87, 5.88] vs. 0.26[0.10, 0.52] µg/mL; *P* < 0.01). Among men, PZP levels were significantly elevated in ACS patients compared to controls (median [IQR]: 0.29[0.12, 0.55] vs. 0.15[0.06, 0.32] µg/mL; *P* < 0.01), whereas no difference was observed in women (median [IQR]: 3.45[1.82, 5.99] vs. 3.48[2.26, 4.89] µg/mL; *P* = 0.95). Correlation analyses revealed distinct sex-specific patterns: in men, PZP correlated positively with age and inversely with estimated glomerular filtration rate (eGFR), whereas in women, only a weak positive correlation with high-density lipoprotein cholesterol (HDL-C) was observed. In multivariable logistic regression, higher PZP was independently associated with ACS in men across all modeling strategies—continuous (fully adjusted OR 5.90, 95% CI 1.59–24.98; *P* = 0.011), dichotomized using the Youden-derived cutoff (> 0.25 µg/mL; OR 1.87, 95% CI 1.02–3.50; *P* = 0.045), and quartile-based (top vs. bottom quartile; OR 4.79, 95% CI 1.74–15.26; *P* = 0.004)—but no significant association was found in women (fully adjusted continuous OR 1.06, 95% CI 0.86–1.31; *P* = 0.589).

**Conclusions:**

This study establishes that circulating PZP exhibits extreme sexual dimorphism and demonstrates its male-specific association with ACS through absolute quantification. Our findings highlight the necessity of sex-stratified analysis in cardiovascular biomarker research. These findings warrant prospective validation to determine whether PZP has any role in sex-stratified cardiovascular risk assessment, as well as future studies to clarify its temporal dynamics and sex-specific mechanisms.

## Abstract (plain language summary)

PZP is a protein that can be measured in the blood. In this study, we investigated whether circulating PZP levels are associated with ACS and whether this association differs between men and women. We measured PZP in hospitalized ACS patients and in non-ACS controls from routine health examinations. We found that PZP levels were much higher in women than in men. Among men, those with ACS had higher PZP levels than controls, whereas among women, PZP levels did not differ based on ACS status. PZP also showed different associations with other clinical measures by sex: in men, higher PZP was linked to older age and lower kidney function; in women, a weak positive correlation with HDL-C was observed. After accounting for other risk factors, higher PZP remained strongly associated with ACS in men but not in women. Together, these findings suggest a male-specific association between PZP and ACS in this case-control setting, underscoring the need to consider sex separately in heart disease research.

## Highlights

Plasma PZP circulates in the µg/mL range and exhibits an extreme sexual dimorphism, with levels approximately 13-fold higher in women than in men.

PZP shows distinct sex-specific correlates: positively associated with age and inversely with eGFR in men, and weakly positively associated with HDL-C in women.

In this hypothesis-generating case-control study, elevated PZP is independently associated with ACS in men after multivariable adjustment, an association not observed in women.

## Introduction

ACS continues to impose a formidable global health burden, remaining a preeminent cause of morbidity and mortality worldwide [1–5]. Despite advancements in acute management and secondary prevention, its epidemiological impact persists [1–5]. Notably, significant sex differences exist across clinical presentation, diagnostic and therapeutic response, and long-term prognosis. Epidemiologically, ACS predominantly affects males, who account for approximately 70% of contemporary ACS admissions [6]. Men also tend to present at a younger age, whereas women generally develop ACS later in life [7]. In women, the incidence rises markedly with age, particularly around the menopausal transition [8, 9]. Additionally, women face a significantly higher long-term risk of adverse cardiovascular events, including recurrent myocardial infarction and mortality, compared to men [10, 11]. The underlying causes of this sex-related clinical heterogeneity remain incompletely understood and may fundamentally arise from intrinsic differences in vascular physiology, hormonal regulation, and metabolic profiles between men and women. However, the traditional “sex-agnostic” approach to biomarker research is increasingly recognized as inadequate for advancing precision medicine.

Several circulating organokines demonstrate significant sex-specific differences in their clinical implications, offering crucial insights for sex-stratified risk assessment and prognosis [12–15]. For instance, elevated fibroblast growth factor 23 shows greater predictive value for adverse outcomes in men, while Klotho protein exhibits an independent protective effect against all-cause mortality exclusively in women [16]. PZP is a highly glycosylated macromolecular protein belonging to the α-macroglobulin family and a fasting-induced hepatokine synthesized predominantly in the liver [17–19], and it is emerging as a multifaceted regulator with pleiotropic functions beyond its canonical role [20]. It promotes diet-induced thermogenesis and energy expenditure by upregulating uncoupling protein 1 in brown adipose tissue [18] and maintains proteostasis by inhibiting the aggregation of misfolded proteins, such as amyloid-beta, through the formation of stable complexes with early oligomers—a function critically upregulated during pregnancy [21]. Clinically, elevated PZP levels in serum exosomes have been identified in patients with inflammatory bowel disease and in murine models of acute colitis, highlighting its potential as a novel serological biomarker [22]. Moreover, proteomic analyses have also identified PZP as a differential circulating protein in early-onset myocardial infarction [23]. However, this association has not been independently validated in broader cardiovascular disease spectra, nor have its sex-specific associations been characterized.

Mass spectrometry-based proteomic profiling of human plasma has consistently identified PZP as one of the most markedly sex-biased circulating proteins [24, 25]. PZP has been reported at concentrations reaching up to ~ 1% of serum albumin levels, and mean circulating PZP is significantly elevated in women compared to men [24, 26, 27]. This striking sexual dimorphism has been corroborated by recent cross-platform benchmarking studies, which confirmed PZP as among the most differentially expressed proteins between sexes on both SomaScan and Olink platforms [28]. However, these foundational discoveries are derived primarily from semi-quantitative assays and have not yet established whether the association between PZP and disease risk is modulated by sex. There is a pressing need for direct evidence defining the absolute quantitative levels of PZP, rigorously characterizing its sex-specific differences, and, most importantly, investigating whether its association with cardiovascular disease pathogenesis exhibits sexual dimorphism.

In this study, we measured absolute PZP concentrations via ELISA in both non-ACS controls and ACS patients. This approach enabled us to preliminarily characterize sex-specific plasma PZP levels and to perform a robust analysis of its association with the presence of ACS, explicitly testing for effect modification by sex.

## Methods

### Study subjects

The ACS patients included in this study were derived from a longitudinal multi-omics cohort as previously described [29]. Detailed protocols regarding patient diagnosis, enrollment, and sample collection have been published previously [29]. Briefly, the cohort prospectively and consecutively enrolled ACS patients who underwent coronary angiography. The diagnosis of ACS was established according to the 2023 European Society of Cardiology Guidelines, based on a comprehensive assessment that includes typical ischemic symptoms, electrocardiographic changes, and dynamic changes in cardiac biomarkers [30]. Each diagnosis was confirmed by at least two independent cardiologists. Patients who were unable to provide informed consent, pregnant women, and those with malignant tumors, acute infectious diseases, or advanced liver diseas were excluded. Malignant tumors were defined as any current or past history of cancer, including individuals who were previously treated with radical therapy but still within a timeframe where they cannot be considered definitively treated. For advanced liver disease, we used the clinical standard of cirrhosis with decompensation, which includes conditions such as ascites, variceal bleeding, hepatic encephalopathy, or documented end-stage liver disease.

Non-ACS control subjects were recruited from individuals undergoing routine health examinations at the Health Examination Center of the Central Hospital Affiliated to Shandong First Medical University. Eligible participants were those who had no known history of ischemic heart disease or cardiovascular or cerebrovascular events (including coronary artery disease, angina, stroke, or peripheral vascular disease), had no record of hospitalization or major surgery within the preceding six months, and were willing to provide written informed consent. Individuals were excluded if they had incomplete examination data, were pregnant, or had acute infectious diseases, malignant tumors, or other major systemic illnesses. This study was approved by the Ethics Committee of the Central Hospital Affiliated to Shandong First Medical University (Approval No. R202306190133) and was performed in compliance with the principles of the Declaration of Helsinki. All participants provided written informed consent.

### Collection of clinical data

Clinical data were collected at enrollment. Demographic characteristics and lifestyle factors, including age, smoking status, and alcohol consumption, were obtained through standardized face-to-face interviews at enrollment. Body weight and height were measured by trained nurses using standard procedures, and body mass index (BMI) was calculated as weight in kilograms divided by height in meters squared (kg/m²). Systolic blood pressure (SBP) and diastolic blood pressure (DBP) were measured by trained nursing staff according to routine clinical practice. Smoking was defined as current smoking of any tobacco products, either daily or occasionally [31, 32]. Alcohol drinking was defined as a weekly alcohol consumption of more than 14 standard units for men and more than 7 standard units for women, with one standard unit equivalent to approximately 10 g of pure alcohol [33]. For all female participants, menopausal status was obtained via a questionnaire that inquired about menstrual cycle patterns, duration, and the date of last menstruation. Based on this information, women were classified as postmenopausal (documented cessation of menses for ≥ 12 consecutive months), premenopausal (ongoing menstruation), or perimenopausal (menstrual irregularity without 12 months of amenorrhea) [34].

Fasting venous blood samples were collected from ACS patients and control participants using a standardized protocol. For ACS patients, samples were obtained in the early morning (after overnight fasting) on the first day following hospital admission. For control participants recruited from the Health Examination Center, fasting venous blood samples were collected in the morning during routine health examinations under identical fasting conditions. The same sampling protocol was applied regardless of sex. Specifically, 4 mL of peripheral blood was collected from the antecubital vein using a sterile vacuum system into anticoagulant tubes. The collected blood was immediately stored at 4 °C and processed within 4 h. Plasma was separated by centrifugation at 3000 rpm for 15 min. All plasma aliquots were initially preserved at − 40 °C and subsequently transferred to − 80 °C freezers within one month for long-term storage. Laboratory parameters, including fasting glucose, glycated hemoglobin (HbA1c), total cholesterol (TC), triglycerides (TG), low-density lipoprotein cholesterol (LDL-C) and HDL-C, uric acid (UA), alanine aminotransferase (ALT), and aspartate aminotransferase (AST), were analyzed using routine biochemical assays in the hospital clinical laboratories.

### Assessment of angiographic severity

Coronary angiography was performed according to routine clinical practice, and angiographic severity was systematically evaluated using the validated Gensini score. The Gensini score was calculated according to American Heart Association guidelines [35] by integrating both stenosis severity and anatomical significance. Briefly, each lesion was assigned a stenosis severity score (1 for 1–25%, 2 for 26–50%, 4 for 51–75%, 8 for 76–90%, 16 for 91–99%, and 32 for total occlusion). The severity score was then multiplied by a location-specific weighting factor reflecting the functional importance of the lesion site: 5× for the left main coronary artery (LMCA); 2.5× for the proximal segments of the left anterior descending artery (LAD) and left circumflex artery (LCx); 1.5× for the mid-segment of the LAD; 1× for the distal segment of the LAD, the mid and distal segments of the LCx, the first diagonal branch (D1), the obtuse marginal branch (OM), the posterior descending branch (PD), and all segments of the right coronary artery (RCA); and 0.5× for the second diagonal branch (D2), third diagonal branch (D3), and posterior lateral branch. The total Gensini score was obtained by summing weighted scores across all lesions.

### Measurement of plasma PZP concentrations

Plasma concentrations of PZP were quantified using a commercially available sandwich ELISA kit (Human PZP DuoSet^®^ ELISA, R&D Systems, Minneapolis, MN, USA; Catalog No. DY8280-05), according to the manufacturer’s instructions. Briefly, 96-well microplates were coated overnight at room temperature with a mouse anti-human PZP capture antibody. After blocking with reagent diluent (1% bovine serum albumin in PBS), plasma samples and recombinant human PZP standards were added in duplicate and incubated for 2 h at room temperature. Following washing, a biotinylated mouse anti-human PZP detection antibody was applied, followed by streptavidin-horseradish peroxidase conjugate. Color development was achieved using tetramethylbenzidine substrate and stopped with sulfuric acid. Optical density was measured at 450 nm with wavelength correction at 540–570 nm. PZP concentrations were calculated from a standard curve generated using a four-parameter logistic (4-PL) regression model. Samples with concentrations exceeding the upper limit of detection were appropriately diluted and re-assayed. The limit of quantification was 156 pg/mL for the PZP ELISA. The intra-assay and inter-assay coefficients of variation for the PZP ELISA were < 8% and < 12%, respectively.

### Statistical analysis

All statistical analyses were performed using R software (version 4.3.3). Continuous variables were assessed for normality. Normally distributed data are presented as mean ± standard deviation (SD) and were compared using the independent-samples t test. Non-normally distributed data are presented as median (interquartile range [IQR], Q1-Q3) and were compared using the Mann-Whitney U test. Categorical variables are expressed as number (n) and percentage (%) and were compared using the χ² test. Spearman rank correlation analyses were used to examine associations between plasma PZP levels and clinical characteristics.

To evaluate the association between plasma PZP concentration and ACS, PZP was modeled in three ways: (1) as a continuous variable to assess an overall dose-response association; (2) as a dichotomous variable using exploratory sex-specific cutoffs determined by the Youden index for internal grouping; and (3) as sex-specific quartiles to explore potential non-linear associations and to compare extreme categories (robustness/sensitivity analysis). Multivariable logistic regression analyses, stratified by sex, were conducted to determine whether plasma PZP was independently associated with the presence of ACS. Covariates for the fully adjusted model were selected based on clinical relevance, their statistical significance in univariable analyses and the disjunctive cause criterion [36]. Three models were constructed: Model 1 was unadjusted and included PZP as the sole independent variable; Model 2 was adjusted for age; and Model 3 was fully adjusted for all covariates that were statistically significant in the univariable analyses, as well as factors considered a priori to influence PZP levels such as menopausal status in females. All statistical tests were two-sided, and a *P* value < 0.05 was considered statistically significant.

## Results

### Demographic and clinical characteristics of subjects included in this study

A total of 1170 subjects were included in this study, comprising 721 males and 449 females. Clinical characteristics, stratified by sex, are shown in Table 1. Compared with males, females were significantly older and had higher levels of HbA1c and TC (all *P* < 0.01). Males exhibited significantly higher BMI, DBP, UA and eGFR (all *P* < 0.01). The prevalence of current smoking and alcohol consumption was markedly higher in males than in females. Medication use was largely comparable between sexes, except for a slightly higher proportion of females using glucose-lowering drugs.

**Table 1:** The demographic and clinical characteristics of all participants stratified by sex

Characteristics	Total (*N* = 1170)	Male (*N* = 721)	Female (*N* = 449)	*P* value
Age, y	62.00 [52.00, 69.00]	60.00 [50.00, 68.00]	65.00 [56.00, 72.00]	< 0.01
BMI, kg/m²	25.39 [23.23, 27.47]	25.77 [23.66, 27.70]	24.80 [22.46, 27.31]	< 0.01
SBP, mmHg	129.50 ± 19.04	129.19 ± 18.50	129.98 ± 19.74	0.50
DBP, mmHg	78.00 [70.00, 88.00]	79.00 [71.00, 89.00]	77.00 [70.00, 84.00]	< 0.01
HbA1c, %	6.10 [5.70, 6.80]	6.00 [5.60, 6.60]	6.10 [5.70, 7.20]	< 0.01
UA, µmol/L	315.00 [266.25, 371.00]	338.00 [288.00, 391.00]	280.00 [234.00, 335.00]	< 0.01
eGFR, mL/min/1.73 m²	91.24 [81.47, 99.36]	92.36 [82.75, 100.28]	89.33 [79.40, 97.27]	< 0.01
HDL-C, mmol/L	1.09 [0.94, 1.29]	1.03 [0.89, 1.20]	1.21 [1.04, 1.44]	< 0.01
LDL-C, mmol/L	2.53 [1.96, 3.05]	2.52 [1.93, 2.99]	2.53 [2.06, 3.14]	0.07
TG, mmol/L	1.36 [1.03, 1.87]	1.37 [1.02, 1.91]	1.35 [1.03, 1.85]	0.61
TC, mmol/L	4.41 [3.77, 5.07]	4.34 [3.64, 4.95]	4.57 [4.03, 5.27]	< 0.01
GLU, mmol/L	5.37 [4.80, 6.42]	5.36 [4.76, 6.30]	5.39 [4.86, 6.64]	0.05
ALT, U/L	20.10 [13.83, 32.40]	22.30 [16.00, 34.70]	17.00 [11.90, 27.50]	< 0.01
AST, U/L	21.45 [16.70, 43.65]	22.40 [17.30, 57.10]	19.70 [15.90, 31.00]	< 0.01
PZP, µg/mL	0.56 [0.18, 2.58]	0.26 [0.10, 0.52]	3.46 [1.87, 5.88]	< 0.01
Smoking, n (%)	438 (37.43)	406 (56.31)	32 (7.10)	< 0.01
Alcohol drinking, n (%)	445 (38.03)	417 (57.84)	28 (6.20)	< 0.01
Hypertension, n (%)	750 (64.10)	448 (62.14)	302 (67.00)	0.08
Diabetes, n (%)	451 (38.55)	259 (35.92)	192 (42.76)	0.02
PCI with stent implantation, n (%)	625 (53.42)	415 (57.56)	210 (46.77)	< 0.01
Medications, n (%)				
Blood pressure-lowering	796 (68.3)	500 (69.35)	296 (65.92)	0.25
Lipid-lowering	941 (80.43)	583 (80.86)	358 (79.73)	0.69
Glucose-lowering	406 (34.70)	231 (32.04)	175 (38.98)	0.02
Antiplatelet	954 (81.54)	590 (81.83)	364 (81.07)	0.80

Normally distributed continuous variables are presented as mean ± SD, whereas non-normally distributed continuous variables are presented as median (interquartile range [IQR]). Categorical variables are presented as n (%). Abbreviations: BMI, body mass index; SBP, systolic blood pressure; DBP, diastolic blood pressure; HbA1c, glycated hemoglobin; TC, total cholesterol; UA, uric acid; eGFR, estimated glomerular filtration rate; HDL-C, high-density lipoprotein cholesterol; LDL-C, low-density lipoprotein cholesterol; TG, triglycerides; GLU, glucose; ALT, alanine aminotransferase; AST, aspartate aminotransferase; PZP, pregnancy zone protein

Clinical characteristics of ACS patients and controls were then evaluated separately by sex (Tables 2 and 3). Among males, ACS patients were significantly older and exhibited higher levels of HbA1c, fasting glucose, ALT, and AST compared to controls (all *P* < 0.05). No significant differences were observed between male ACS patients and controls in TC, TG and LDL-C. The prevalence of smoking, alcohol consumption, hypertension, and diabetes was also significantly higher in male ACS patients (all *P* < 0.01). In females, ACS patients were older and had higher BMI compared to controls. They also exhibited significant disturbances in glucose and lipid metabolism, as well as higher SBP and DBP. Additionally, female ACS patients showed elevated UA, ALT, and AST levels, accompanied by lower eGFR (all *P* < 0.01). In contrast to males, the proportions of current smoking and alcohol drinking did not differ significantly between female ACS patients and controls.

**Table 2:** The demographic and clinical characteristics of ACS patients and control subjects

Characteristics	Male			Female			Male vs. Female controls	Male vs. Female ACS
	Control (*N* = 102)	ACS patients (*N* = 619)	*P* value	Control (*N* = 67)	ACS patients (*N* = 382)	*P* value	*P* value	*P* value
Age, y	47.00 [39.25, 53.00]	62.00 [54.00, 68.50]	< 0.01	42.00 [36.00, 47.50]	67.00 [60.00, 72.00]	< 0.01	0.01	< 0.01
BMI, kg/m²	25.52 [23.60, 27.13]	25.95 [23.88, 27.90]	0.29	22.69 [21.50, 25.31]	24.98 [22.86, 27.34]	< 0.01	< 0.01	< 0.01
SBP, mmHg	127.43 ± 17.70	129.52 ± 18.70	0.28	114.76 ± 17.60	132.66 ± 18.90	< 0.01	< 0.01	0.03
DBP, mmHg	80.00 [74.00, 87.00]	79.00 [70.00, 89.00]	0.46	69.00 [62.50, 78.00]	78.00 [71.00, 85.00]	< 0.01	< 0.01	0.14
HbA1c, %	5.90 [5.50, 6.20]	6.10 [5.60, 6.80]	< 0.01	5.80 [5.50, 6.00]	6.25 [5.80, 7.30]	< 0.01	0.39	< 0.01
UA, µmol/L	353.00 [319.00, 384.50]	331.00 [284.00, 390.00]	0.02	254.00 [222.00, 288.50]	282.00 [235.00, 339.00]	< 0.01	< 0.01	< 0.01
eGFR, mL/min/1.73 m²	96.47 [87.39, 103.65]	92.01 [82.45, 99.70]	< 0.01	103.53 [94.43, 111.05]	87.95 [74.53, 94.74]	< 0.01	< 0.01	< 0.01
HDL-C, mmol/L	1.17 [1.00, 1.39]	1.01 [0.87, 1.17]	< 0.01	1.39 [1.23, 1.62]	1.18 [1.02, 1.39]	< 0.01	< 0.01	< 0.01
LDL-C, mmol/L	2.42 ± 0.50	2.51 ± 0.80	0.15	2.32 [1.94, 2.49]	2.63 [2.10, 3.27]	< 0.01	0.01	0.01
TG, mmol/L	1.33 [1.00, 1.84]	1.40 [1.03, 1.93]	0.13	0.95 [0.66, 1.31]	1.42 [1.10, 1.92]	< 0.01	< 0.01	0.33
TC, mmol/L	4.29 ± 0.60	4.36 ± 1.00	0.37	4.29 [4.02, 4.54]	4.71 [4.05, 5.45]	< 0.01	0.44	< 0.01
GLU, mmol/L	5.13 [4.78, 5.67]	5.44 [4.76, 6.50]	0.02	4.93 [4.69, 5.30]	5.64 [4.92, 6.96]	< 0.01	0.11	< 0.01
ALT, U/L	20.15 [14.77, 28.27]	22.90 [16.20, 36.40]	0.04	12.30 [9.35, 16.90]	17.50 [12.22, 28.58]	< 0.01	< 0.01	< 0.01
AST, U/L	19.15 [16.00, 22.37]	24.00 [17.50, 73.40]	< 0.01	16.10 [14.25, 19.8]	20.95 [16.30, 35.60]	< 0.01	< 0.01	< 0.01
PZP, µg/mL	0.15 [0.06, 0.32]	0.29 [0.12, 0.55]	< 0.01	3.48 [2.26, 4.89]	3.45 [1.82, 5.99]	0.95	< 0.01	< 0.01
Smoking, n (%)	28 (27.45)	379 (61.23)	< 0.01	1 (1.49)	31 (8.12)	0.07	< 0.01	< 0.01
Alcohol drinking, n (%)	47 (46.08)	374 (60.42)	< 0.01	4 (5.97)	27 (7.07)	0.80	< 0.01	< 0.01
Hypertension, n (%)	20 (19.61)	430 (69.47)	< 0.01	4 (5.97)	299 (78.27)	< 0.01	0.07	< 0.01
Diabetes, n (%)	9 (8.82)	251 (40.55)	< 0.01	4 (5.97)	190 (49.74)	< 0.01	0.77	0.01
PCI with stent implantation, n (%)	0 (0)	415 (67.04)	-	0 (0)	210 (54.97)	-	-	< 0.01
Medications, n (%)								
Blood pressure-lowering	0 (0)	500 (80.78)	-	0 (0)	296 (77.49)	-	-	0.21
Lipid-lowering	0 (0)	583 (94.18)	-	0 (0)	358 (93.72)	-	-	0.77
Glucose-lowering	0 (0)	231 (37.32)	-	0 (0)	175 (45.81)	-	-	< 0.01
Antiplatelet	0 (0)	590 (95.32)	-	0 (0)	364 (95.29)	-	-	0.98

Normally distributed continuous variables are presented as mean ± SD, whereas non-normally distributed continuous variables are presented as median (interquartile range [IQR]). Categorical variables are presented as n (%). Abbreviations are the same as those used in Table 1

**Table 3:** Univariable logistic regression analysis of clinical characteristics associated with ACS in male and female participants

Characteristics	Male			Female		
	OR	95% CI	*P* value	OR	95% CI	*P* value
Age, y	1.12	(1.10,1.15)	< 0.01	1.28	(1.22,1.36)	< 0.01
BMI, kg/m²	1.02	(0.95,1.08)	0.64	1.17	(1.08,1.28)	< 0.01
SBP, mmHg	1.00	(0.99,1.02)	0.41	1.06	(1.04,1.08)	< 0.01
DBP, mmHg	0.99	(0.98,1.01)	0.40	1.05	(1.03,1.08)	< 0.01
HbA1c, %	1.64	(1.27,2.19)	< 0.01	2.45	(1.68,3.84)	< 0.01
UA, µmol/L	1.00	(0.99,1.00)	< 0.01	1.01	(1.00,1.01)	< 0.01
eGFR, mL/min/1.73 m²	0.97	(0.96,0.99)	< 0.01	0.89	(0.86,0.92)	< 0.01
HDL-C, mmol/L	0.10	(0.04,0.21)	< 0.01	0.12	(0.05,0.27)	< 0.01
LDL-C, mmol/L	1.17	(0.88,1.55)	0.29	2.02	(1.41,2.94)	< 0.01
TG, mmol/L	1.32	(1.01,1.81)	0.06	2.85	(1.72,5.00)	< 0.01
TC, mmol/L	1.07	(0.85,1.35)	0.55	1.72	(1.28,2.36)	< 0.01
GLU, mmol/L	1.24	(1.08,1.46)	< 0.01	1.64	(1.30,2.17)	< 0.01
ALT, U/L	1.01	(1.00,1.02)	0.02	1.06	(1.03,1.10)	< 0.01
AST, U/L	1.04	(1.02,1.06)	< 0.01	1.07	(1.03,1.12)	< 0.01
PZP, µg/mL	13.32	(4.72,37.60)	< 0.01	1.04	(0.94,1.15)	0.46
Smoking	4.80	(3.01,7.88)	< 0.01	6.03	(1.26,108.28)	0.08
Alcohol drinking	1.68	(1.11,2.57)	0.02	1.05	(0.39,3.68)	0.92
Hypertension	10.62	(6.35,18.69)	< 0.01	76.85	(27.62,319.98)	< 0.01
Diabetes	8.01	(4.07,18.19)	< 0.01	21.33	(7.74,88.33)	< 0.01

Continuous variables are per unit increase. Categorical variables are compared to the reference category. Abbreviations are the same as those used in Table 1

### Sex differences in plasma PZP concentrations between control subjects and ACS patients

**Fig. 1:**
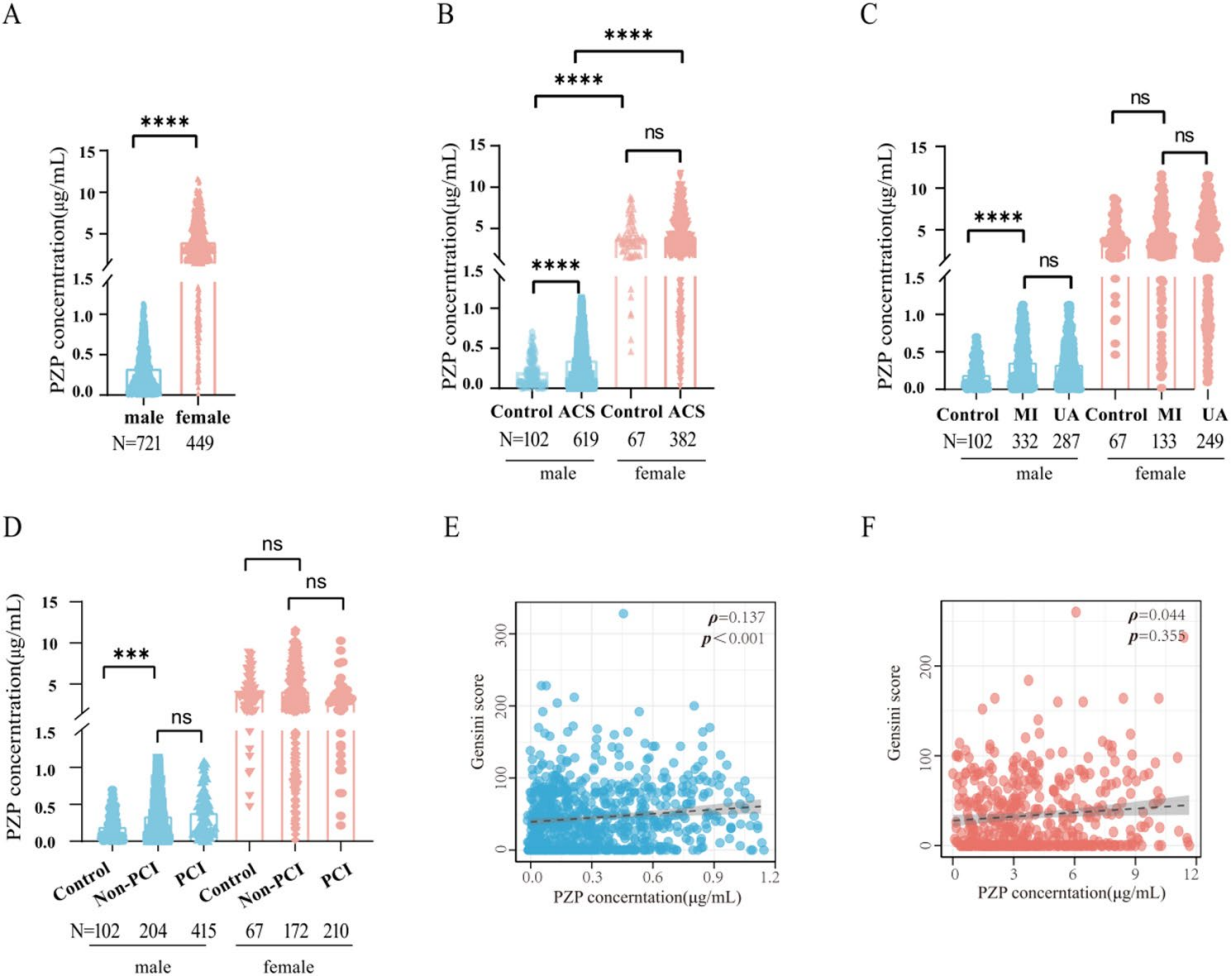
Sex-stratified Comparison of Plasma PZP Levels and Their Correlation with Coronary Severity. (**A**) PZP concentrations in male and female participants. (**B**) PZP concentrations in controls and ACS patients, stratified by sex. (**C**) Plasma PZP concentrations in controls and ACS subtypes (MI and UA), stratified by sex. (**D**) Plasma PZP concentrations in controls and patients with or without PCI treatment, stratified by sex. (E-F) Scatter plots depict sex-stratified correlations between plasma PZP concentration and Gensini score in males (**E**) and females (**F**). Individual data points are overlaid for each group and sample sizes are indicated below the corresponding bars. Between-group comparisons within each sex were assessed using the two-tailed Mann-Whitney U test. An axis break is used to accommodate the markedly different PZP concentration ranges between sexes. For correlation analyses, the correlation coefficient (*ρ*) and *P* value are shown in each panel, and the fitted trend line with 95% confidence interval is displayed. ****, *P* < 0.0001; ns, not significant

We next evaluated plasma PZP levels with respect to both sex-specific differences and case-control status. As anticipated from prior proteomic surveys, a profound sexual dimorphism was observed, with PZP concentrations markedly higher in women than in men (median: 3.46 µg/mL in females vs. 0.26 µg/mL in males, Table 1; Fig. 1A). This represents an approximately 13-fold sex-based difference, conclusively establishing PZP as a highly sex-biased plasma protein. When stratified by case-control status, PZP levels did not differ between ACS patients and controls in females (3.45 vs. 3.48 µg/mL; *P* = 0.95; Table 2; Fig. 1B). By contrast, among males, PZP concentrations were significantly elevated in ACS patients compared to controls (0.29 vs. 0.15 µg/mL; *P* < 0.0001), corresponding to a 1.9-fold increase. Notably, female PZP levels remained substantially higher than male levels within both the control and ACS groups (Fig. 1B).

Further subgroup analyses revealed that, in males, PZP concentrations were significantly higher in both MI and UA patients than in controls (both *P* < 0.0001), and similarly elevated in ACS patients irrespective of PCI treatment (both *P* < 0.001 vs. controls; Fig. 1C-D). In females, however, no significant differences were observed between any of these subgroups and controls (Fig. 1C-D). To further examine the association between circulating PZP levels and the burden of coronary lesions, we examined their correlation with Gensini scores (Fig. 1E-F). Spearman correlation analyses showed a weak but statistically significant correlation between PZP and Gensini score in males (*ρ* = 0.137, *P* < 0.001), but not in females (*ρ* = 0.044, *P* = 0.355). Taken together, these results provide the first absolute quantification of PZP in a cardiovascular context, confirming its high abundance in the microgram-per-milliliter range and its extreme sexual dimorphism. Importantly, a male-specific association between circulating PZP and ACS status was detected after disease onset, but not in women, highlighting the necessity of sex-stratified analysis when investigating its association with ACS.

### Sex-specific associations between plasma PZP and clinical variables

**Fig. 2:**
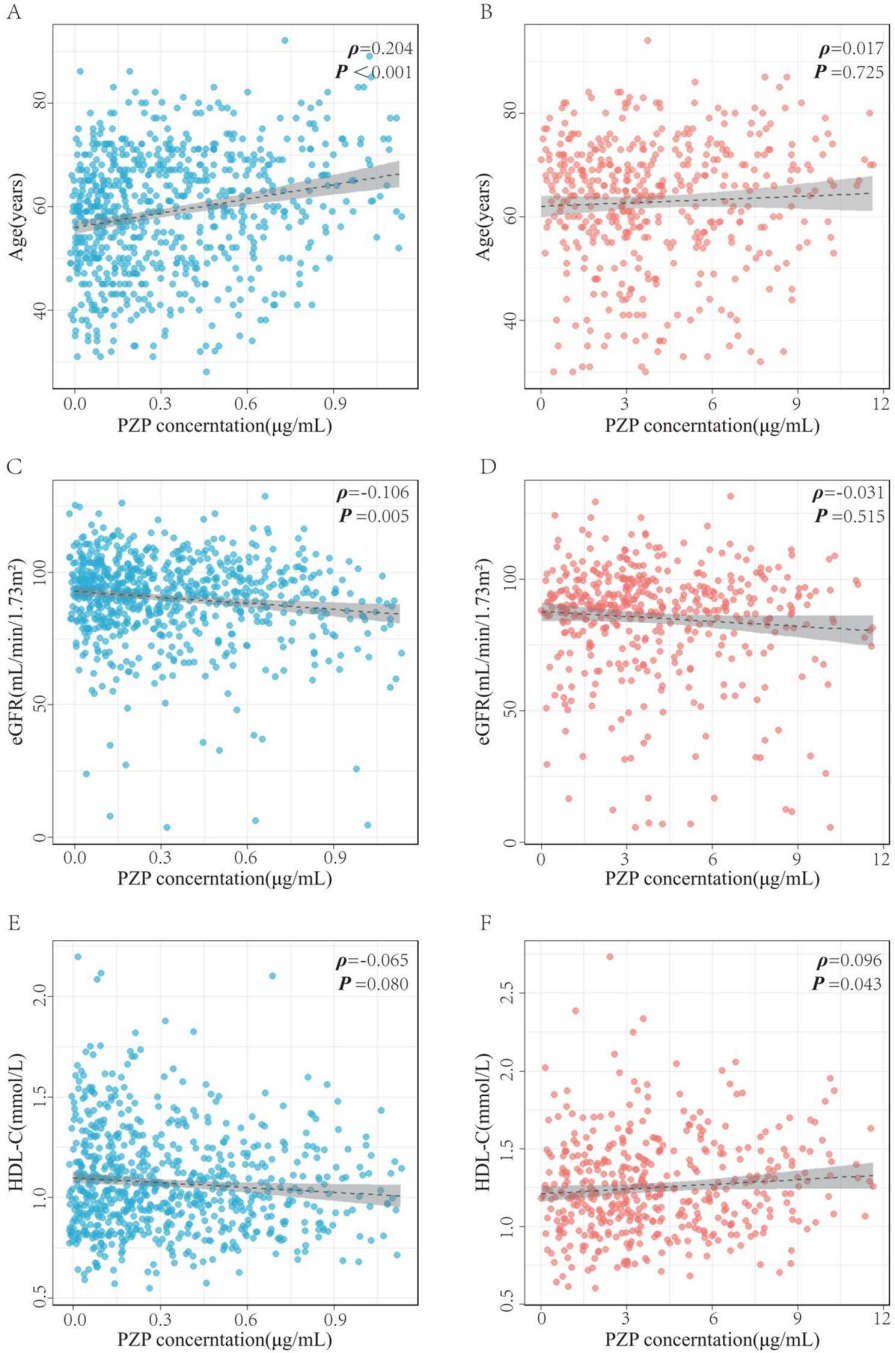
Sex-specific Associations of Plasma PZP Concentration with the Clinical Variables. Scatter plots depict the correlations in male (**A**, **C**, **E**) and female (**B**, **D**, **F**) participants, respectively. (**A**-**B**) Correlation between plasma PZP concentration and age. (**C**-**D**) Correlation between plasma PZP concentration and eGFR. (**E**-**F**) Correlation between plasma PZP concentration and HDL-C. Spearman’s correlation coefficient (*ρ*) and *P* value are reported in each panel; the dashed line indicates the fitted trend. Each dot represents one participant

To characterize the sex-specific clinical correlates of plasma PZP, we performed Spearman rank correlation analyses separately for males and females (Table 4; Fig. 2). Different correlation patterns were observed between sexes. In males, PZP levels were positively correlated with age (*ρ* = 0.204, *P* < 0.001) and inversely correlated with eGFR (*ρ* = -0.106, *P* = 0.005), whereas these associations were absent in females (age: *ρ* = 0.017, *P* = 0.725; eGFR: *ρ* = -0.031, *P* = 0.515, Fig. 2A-D). In females, a weak positive correlation was observed between PZP and HDL-C (*ρ* = 0.096, *P* = 0.043, Fig. 2F), an association not evident in males (*ρ* = -0.065, *P* = 0.080, Fig. 2E). These results indicate that the clinical correlates of plasma PZP differ significantly between sexes. Accordingly, sex-specific covariates were selected for adjustment in subsequent multivariable regression models to account for this critical biological variable.

**Table 4:** Spearman rank correlation analysis of PZP with clinical variables

Characteristics	Male		Female	
	ρ	*P* value	ρ	*P* value
Age	0.204	< 0.001*	0.017	0.725
BMI	0.053	0.152	-0.004	0.937
SBP	0.002	0.956	0.037	0.429
DBP	-0.067	0.073	-0.009	0.850
HbA1c	0.062	0.098	0.014	0.762
TC	-0.054	0.150	0.016	0.729
UA	-0.041	0.276	-0.025	0.603
eGFR	-0.106	0.005*	-0.031	0.515
HDL-C	-0.065	0.080	0.096	0.043*
LDL-C	-0.010	0.795	0.012	0.801
TG	-0.033	0.377	-0.023	0.624
GLU	0.035	0.343	-0.027	0.571
ALT	-0.004	0.922	-0.065	0.170
AST	0.012	0.744	-0.073	0.122
Smoking	0.058	0.122	-0.003	0.949
Alcohol drinking	-0.027	0.471	-0.024	0.610

Spearman correlation coefficients (*ρ*) and corresponding *P* values are presented. A *P* value < 0.05 was considered statistically significant. * indicates *P* < 0.05

### Sex-specific association between plasma PZP concentration and ACS

**Fig. 3:**
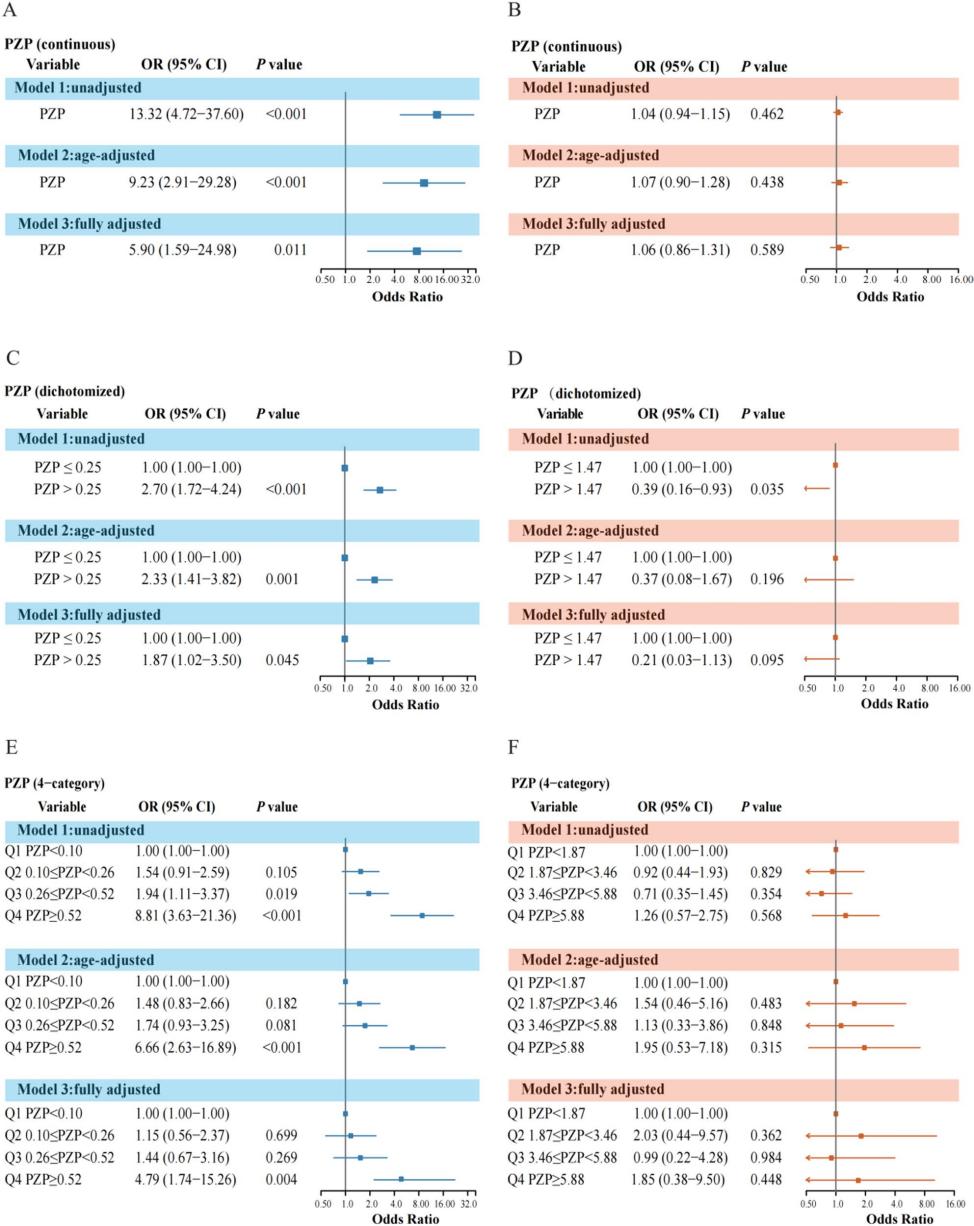
Sex-specific Association between Plasma PZP and the Presence of ACS. Forest plot showing odds ratios (ORs) and 95% confidence intervals (CIs) from sex-stratified logistic regression analyses in men (**A**, **C**, **E**) and women (**B**, **D**, **F**). (**A-B**) PZP modeled as a continuous variable. (**C-D**) PZP modeled as a dichotomous variable using sex-specific optimal cutoffs derived from the Youden index (men: 0.25 µg/mL; women: 1.47 µg/mL). (**E-F**) PZP modeled as sex-specific quartiles (men: Q1 < 0.10, Q2 0.10–0.26, Q3 0.26–0.52, Q4 ≥ 0.52 µg/mL; women: Q1 < 1.87, Q2 1.87–3.46, Q3 3.46–5.88, Q4 ≥ 5.88 µg/mL). Three models are displayed: Model 1 (unadjusted), Model 2 (adjusted for age), and Model 3 (fully adjusted). In men, Model 3 was adjusted for age, HbA1c, UA, eGFR, HDL‑C, glucose, ALT, AST, smoking, alcohol consumption, and the use of blood pressure-lowering, lipid-lowering, glucose-lowering, and antiplatelet medications. In women, Model 3 was adjusted for age, BMI, SBP, DBP, HbA1c, TC, UA, eGFR, HDL‑C, LDL‑C, TG, glucose, ALT, AST, menopausal status, and the use of blood pressure-lowering, lipid-lowering, glucose-lowering, and antiplatelet medications. Squares represent point estimates; horizontal lines denote 95% CIs. Arrows indicate confidence intervals extending beyond the plotted axis range

We employed sex-stratified logistic regression models to evaluate the independent association of plasma PZP with ACS. Plasma PZP was analyzed both as a continuous variable, to assess its overall association with ACS, and as a categorical variable to enhance clinical interpretability (Fig. 3). In males, higher PZP levels were associated with increased odds of ACS when modeled as a continuous variable, with an unadjusted OR of 13.32 (95% CI: 4.72–37.60; *P* < 0.001). The association remained significant in the fully adjusted model (OR 5.90, 95% CI: 1.59–24.98; *P* = 0.011). No significant association was observed in females (fully adjusted model: OR 1.06, 95% CI: 0.86–1.31; *P* = 0.589).

Categorical analyses were consistent with the findings observed in males. When dichotomized at the Youden-derived cutoff of 0.25 µg/mL, elevated PZP remained significantly associated with higher odds of ACS in males across all models, including the fully adjusted model (OR 1.87, 95% CI 1.02–3.50; *P* = 0.045). In females, a nominal inverse association observed in the unadjusted dichotomous analysis did not remain significant after multivariable adjustment. Similarly, in quartile-based analysis, males in the highest PZP quartile exhibited significantly elevated ACS odds compared to the lowest quartile, an association that persisted after full adjustment (OR 4.79, 95% CI 1.74–15.26; *P* = 0.004). Consistently, no significant associations across PZP quartiles were observed in females in any model (*P* > 0.05). Collectively, these sex-stratified regression analyses suggest that the association between plasma PZP and ACS was observed in males but not in females in this dataset.

## Discussion

In this study, we examined the association between plasma PZP and ACS with a specific emphasis on sex as a biological variable. Our findings provide the first report of absolute PZP quantification in a cardiovascular context, indicating that PZP circulates at relatively high levels in the microgram-per-milliliter range and showing marked sex differences in circulating PZP levels—with concentrations approximately 13-fold higher in women than in men. Most importantly, elevated PZP levels were observed in association with ACS in men, even after full multivariable adjustment, whereas no significant association was observed in women. Collectively, these results highlight the potential importance of sex-stratified analysis when evaluating circulating biomarkers, rather than relying on pooled, unstratified evaluations.

This study, comprising over one thousand subjects, provides the most extensive clinical evidence to date on the sex-specific characteristics of plasma PZP. A key methodological strength is the use of a commercial ELISA kit to obtain absolute protein concentrations. Moving beyond the relative quantifications typical of exploratory proteomic screens, this approach yields values in microgram-per-milliliter units, which are essential for direct cross-study comparisons and for grounding biomarker discussions in clinically interpretable metrics. Our data suggest pronounced sexual dimorphism of PZP, with absolute circulating levels lower in males than in females. Proteomic analyses have previously identified PZP as a differentially expressed protein in patients with early-onset myocardial infarction [23], and population studies have linked it to cardiovascular risk phenotypes such as vascular stiffness [37]. Our study significantly extends this evidence by demonstrating sex-stratified patterns in the association between PZP and ACS. Specifically, we observed that PZP levels were significantly elevated in males with ACS compared to male controls, an association that persisted after comprehensive multivariable adjustment, including factors such as renal function and medication use history. This phenomenon was not observed in women. Despite these findings, our data do not support immediate clinical application but rather emphasize the importance of sex-stratified analysis in biomarker discovery to avoid obscuring biologically meaningful signals.

The observed elevation of PZP in male ACS patients, coupled with its independent association with ACS risk and positive correlation with Gensini score, suggests a link with disease severity beyond a nonspecific inflammatory response. Several biological functions of PZP have been described in the literature, including anti-protease and immunomodulation activity via receptors such as LRP1 [17], modulation of energy metabolism [18], regulation of fibrinolysis through tissue-type plasminogen activator clearance [38, 39], involvement in neutrophil extracellular trap biology [40], modulation of T-cell responses [41] and a potential role in cellular senescence [42]. In addition, PZP exerts immunomodulatory effects through co-localization with Glycodelin A (GdA) in the decidual stroma, and its abnormal upregulation, particularly when coupled with GdA downregulation, is associated with spontaneous and recurrent miscarriage [43]. While these mechanisms are well-documented in other biological contexts, their specific relevance to ACS pathophysiology has not been directly tested. Sex-specific coronary plaque biology may also provide a framework for interpreting our findings. The CLIMA study demonstrated that plaque vulnerability in women is primarily driven by inflammation and stenosis, whereas in men it is driven by lipid burden [44]. We propose that the male-specific association between PZP and ACS might reflect its involvement in lipid-driven pro-atherogenic processes. This hypothesis remains speculative and requires direct testing in future studies that link circulating PZP levels to specific plaque morphologies using intracoronary imaging.

The coexistence of markedly higher PZP levels in women and a higher incidence of ACS in men [7, 45–48]—while PZP is associated with ACS only in males—requires careful interpretation. First, the observed sexual dimorphism in PZP levels could be explained by multiple, non-mutually exclusive mechanisms, such as hormonal regulation, particularly estrogenic effects, or anatomical and physiological differences, such as differences in body composition. Although previous studies have linked PZP expression to sex hormone pathways, the contribution of other factors cannot be ruled out. Nevertheless, PZP concentration should not be equated with pathophysiological directionality. In men, where physiological concentrations of PZP are substantially lower, the observed elevation in ACS may reflect ACS-associated systemic responses or a hypothesis-generating biological signal, potentially mediated by impaired fibrinolysis via t-PA clearance, NET-associated inflammation, and cellular senescence—pathways previously implicated in atherothrombosis. The absence of a significant elevation in female ACS patients may therefore reflect a ceiling effect due to constitutively high levels, or alternatively, sex-dependent differences in upstream regulatory pathways governing PZP expression. Importantly, we do not propose that lower PZP levels explain the higher incidence of ACS in men, nor do we infer causality. Rather, our findings suggest that the regulatory role and pathophysiological relevance of PZP may differ between sexes, highlighting the necessity of sex-stratified approaches in biomarker research and precision medicine.

Several limitations of this study should be considered when interpreting the findings. First, although we observed a sex-stratified association between PZP and ACS, the case-control design precludes determination of temporality or causal inference. Blood samples were collected after ACS onset; therefore, it remains unknown whether elevated PZP levels precede the development of ACS. As such, these findings should be interpreted as hypothesis-generating. Prospective cohort studies with serial sampling are warranted to establish the temporal relationship between PZP elevation and ACS onset, and mechanistic investigations are needed to elucidate the underlying biological pathways. Second, the non-ACS control subjects were recruited from routine health examinations. These two groups therefore differ not only in the presence of ACS but also in multiple clinical characteristics. Although we performed sex-stratified analyses and adjusted for available covariates in the multivariable models, we acknowledge that statistical adjustment cannot fully overcome this structural imbalance between cases and controls. Third, current data cannot distinguish whether the sexual dimorphism in PZP levels reflects hormonal regulation, sex-based anatomical/physiological differences, or their interaction. Future studies—including transgenic mouse models or specifically designed human cohort studies that incorporate detailed assessments of hormonal status and body composition—are needed to disentangle these possibilities. Fourth, the single-center nature of the study may limit the generalizability of our results to other populations or healthcare settings. The Youden-derived dichotomised PZP cut-offs in this study are data-driven and external validation in larger, independent, multi-center prospective cohorts using standardized ELISA protocols is required before they are considered as definitive clinical thresholds.

## Conclusions

In conclusion, our findings identify PZP as a highly sexually dimorphic protein associated with ACS specifically in men, offering insights into the molecular basis of sex disparities in cardiovascular disease. This underscores the need to integrate sex as a fundamental biological variable in biomarker research. Moving forward, prospective validation and mechanistic studies of PZP’s role will be important to determine whether PZP has any role in sex-stratified cardiovascular risk assessment.

## Data Availability

The datasets generated and analyzed during the current study are available from the corresponding author on reasonable request.
